# Geometric distortions of diffusion weighted imaging of the head/neck in combined PET/MR: optimization of image acquisition and post-processing correction for oncology applications

**DOI:** 10.1186/2197-7364-1-S1-A76

**Published:** 2014-07-29

**Authors:** Adam E Hansen, Jacob Rasmussen, Helle H Johannesen, Astrid M Engberg, Flemming L Andersen, Lena Specht, Andreas Kjaer, Liselotte Højgaard, Thomas Beyer, Barbara M Fischer

**Affiliations:** Dept. of Clinical Physiology, Nuclear Medicine and PET, Rigshospitalet, Copenhagen, Denmark; Dept. of Oncology, Section of Radiotherapy, Rigshospitalet, Copenhagen, Denmark; Centre for Medical Physics and Biomedical Engineering, Medical University Vienna, Kragujevac, Austria

The combination of PET with Diffusion Weighted Imaging (DWI) is a promising application of PET/MR. DWI geometric accuracy can be compromised in body regions with complex anatomy. Here, we assess DWI head/neck image quality following optimization of acquisition and post-processing.

Preliminary data from 10 patients with cancer of the tonsil or base-of-tongue is presented. An integrated PET/MR system (Siemens Biograph mMR) with a 3 T magnet was used.

PET was performed as a single-bed, 20 min acquisition, 120 min post injection of 4 MBq/kg [^18^F]-FDG.

MRI included axial T2 weighted STIR, B0 mapping and 2 DWI single-shot EPI measurements (DWI1 and DWI2) with b values 0, 500 and 1000 mm^2^/s. DWI2 was measured as 3 stacks, each at isocenter to maximize field homogeneity. DWI1/DWI2 had an effective echo spacing 0.375/0.145 ms. For DWI2, distortions were corrected using FSL with FUGUE and Topup algorithms.

DWI geometric quality was evaluated in 44×44 mm^2^ region centred on the tumor, with the STIR image as anatomical reference. Correlation and DICE coefficients between DWI b0 and STIR images were computed, for all DWI image sets.

Geometric quality of DWI1 was poor, but improved both by optimization of acquisition (DWI2) and by post-processing. Visually, hyperintensities on the Topup corrected DWI2 images matched closely the STIR images. Also, regions with high FDG uptake and low diffusion had matching shapes. Correlation and DICE coefficients had a large variation for DWI1, and showed an overall increase to a high level >0.6 and 0.8, respectively, following optimization of image acquisition and post-processing correction.

Diffusion weighted images of the head/neck with a quality suitable for dual-modality assessment of tumour characteristics on a voxel basis can be obtained following the optimization of acquisition and post-processing.

